# Comprehensive proteomic profiling of serum extracellular vesicles in patients with colorectal liver metastases identifies a signature for non-invasive risk stratification and early-response evaluation

**DOI:** 10.1186/s12943-022-01562-4

**Published:** 2022-04-01

**Authors:** Kuailu Lin, Franziska Baenke, Xixi Lai, Martin Schneider, Dominic Helm, Heike Polster, Venkatesh S. Rao, Nicole Ganig, Fang Cheng Wong, Lena Seifert, Adrian M. Seifert, Beatrix Jahnke, Nicole Kretschmann, Tjalf Ziemssen, Fee Klupp, Thomas Schmidt, Martin Schneider, Yi Han, Tim F. Weber, Verena Plodeck, Heiner Nebelung, Nathalie Schmitt, Felix Korell, Bruno C. Köhler, Carina Riediger, Jürgen Weitz, Nuh N. Rahbari, Christoph Kahlert

**Affiliations:** 1 Department of Visceral, Thoracic and Vascular Surgery, Carl Gustav Carus University Hospital Dresden, Technische Universität Dresden, Fetscherstr. 74, 01307 Dresden, Germany; 2 Department of Breast Surgery, The First Affiliated Hospital, College of Medicine, Zhejiang University, Hangzhou, China; 3 German Cancer Research Center (DKFZ), German Cancer Consortium (DKTK), Partner Site Dresden, Heidelberg, Germany; 4 Department of Respiratory and Critical Care Medicine, College of Medicine, Sir Run Run Shaw Hospital, Zhejiang University, Hangzhou, China; 5 MS-Based Protein Analysis Unit, German Cancer Research Centre (DKFZ), Heidelberg, Germany; 6 National Center for Tumor Diseases (NCT/UCC), Partner Site Dresden, Heidelberg, Germany; 7 MS Center, Centre of Clinical Neuroscience, Department of Neurology, Carl Gustav Carus University Hospital Dresden, Dresden, Germany; 8 Department of General, Visceral and Transplantation Surgery, University Hospital Heidelberg, Heidelberg, Germany; 9 Klinik Für Allgemein, Viszeral-, Tumor- Und Transplantationschirurgie, Universitätklinikum Köln, Kerpener Str. 62, 50937 Köln, Germany; 10 Diagnostic and Interventional Radiology (DiR), Heidelberg University Hospital, Im Neuenheimer Feld 420, 69120 Heidelberg, Germany; 11 Department of Diagnostic and Interventional Radiology, Carl Gustav Carus University Hospital Dresden, Dresden, Germany; 12 Department of Medical Oncology, National Center for Tumour Diseases, Liver Cancer Centre Heidelberg, University Hospital Heidelberg, University Hospital Heidelberg, Heidelberg, Germany; 13 Department of Medicine V, University of Heidelberg, Heidelberg, Germany; 14 German Cancer Consortium (DKTK), 69120 Heidelberg, Germany; 15 Department of Surgery, Medical Faculty Mannheim, University Medicine Mannheim, University of Heidelberg, 68167 Mannheim, Germany

**Keywords:** Extracellular vesicles, Protein signature, Molecular risk stratification, Early response prediction, Colorectal liver metastases

## Background

Preoperative risk stratification and chemotherapy-response prediction for patients with colorectal liver metastases (CRLM) remain areas of unmet clinical need. Patients with colorectal liver metastases (CRLM) have a 5-year overall survival (OS) of 25.2% compared to 75.1% of patients without metastases [1]. Surgical resection of CRLM is an established treatment extending 5-year OS to 40–50% [2].

At present, molecular risk stratification of CRLM is based on histopathological assessment and prevalent drivers of disease derived from a tumour biopsy. Liquid biopsies, i.e. non-invasive analyses of circulating tumour-derived material, may provide unique information about the disease, and moreover, allow monitoring of tumour evolution in response to treatment [3].

Circulating extracellular vesicles (cEVs) are commonly found in body fluids. cEVs encapsulate various cargoes, including proteins, lipids and nucleic acids recapitulating molecular traits of its donor tissue [4]. Mutational changes frequently observed in cancer were detected in cEVs of CRC patients on mRNA level [5]. A differential abundance for distinct non-coding RNAs in cEVs in CRC compared to patients with benign disease have also been reported [6–8]. cEVs have been suggested to be more stable compared to serological proteins as the lipid bilayer protects the content from proteases and other enzyme [9]. In contrast to solid tumour biopsies, that are sampled from a single site, cEVs may provide unique information about the full metastatic complement [10].

The aim of this study was to identify a predictive signature based on the protein cargo of circulating extracellular vesicles (cEVs) and to validate this EV protein (EVP) signature in independent patient cohorts.

## Results and discussion

### EVP signature for prognostic prediction CRLM survival

**Fig. 1:**
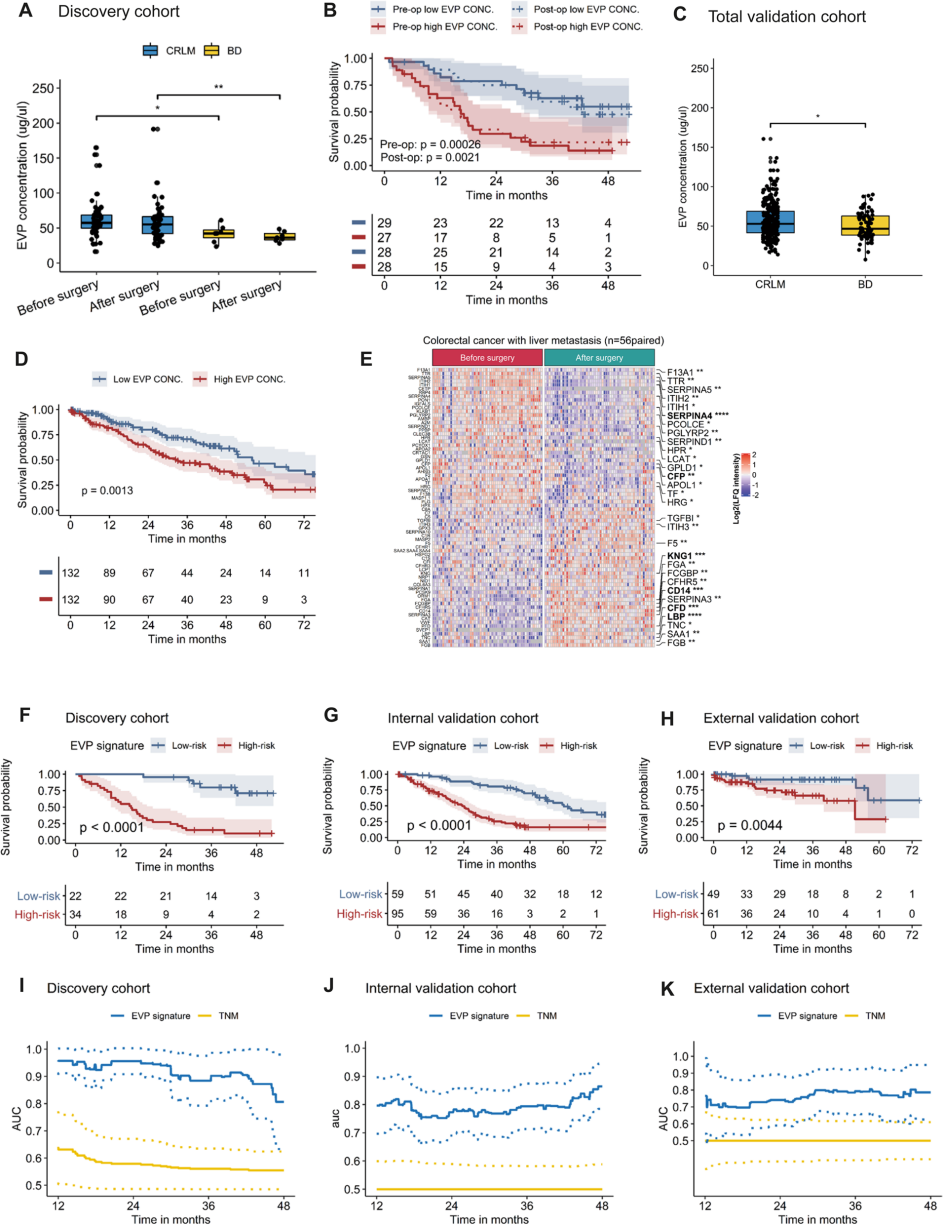
EVP signature for prognostic prediction CRLM survival. Quantification of EVPs from human serum samples **A** Boxplot analysis of EVP concentration in the discovery cohort. *: *p* ≤ 0.05, **: *p* ≤ 0.01. *p* values by Mann-Whitney U test. **B** Kaplan-Meier curve of overall survival for patients with EVP concentration before and after surgery in the discovery cohort. *p* values of two equal-sized parts according to the median of EVP concentration by log-rank test. **C** Boxplot analysis of EVP concentration in the total validation cohort including internal and external validation cohorts. *: *p* ≤ 0.05. p values by Mann-Whitney U test. **D** Kaplan-Meier curve of overall survival for patients with EVP concentration in the total validation cohort. p values of two equal-sized parts according to the median of EVP concentration by log-rank test. **E** Heatmap of 74 differentially expressed EVPs ( FDR<0.05 ). Each column represents an individual patient ( n_paired_ = 56 ) and each row represents an EVP. Labels of the right side represent pre-surgical survival associated proteins by Univariate cox analysis. *: *p* ≤ 0.05, **: *p* ≤ 0.01, ***: *p* ≤ 0.001, ****: *p* ≤ 0.0001. Bold labels are EVPs selected for generating an EVP signature. **F**-**K** EVP analysis for prognostic prediction CRLM survival: *Top panel: *Kaplan-Meier survival analysis was used to analyze survival of low-risk and high-risk groups based on the cut-off value of the risk score estimated from the discovery cohort. **F** Discovery cohort. **G** Internal validation cohort. **H** External validation cohort. *p* values by log-rank test. *Bottom panel: Time-dependent *Receiver operator characteristic (ROC) curves for the prognostic performance of the EVP signature in discovery cohort (**I**), internal validation cohort (**J**) and external validation cohort (**K**). 95%CI of AUC was marked by dotted line

Our discovery cohort (Table 1) included patients with CRLM (*n* = 56) or benign liver disease (*n* = 7; BD) and we observed that the EVP concentration in the serum of the CRLM patients was significantly increased in comparison to patients with BD (before surgery: *p* = 0.01; after surgery: *p* < 0.001; Fig. 1A). Univariate Cox regression analysis revealed that EVP concentration was negatively associated with OS in patients with CRLM pre- and post-surgically (*p* < 0.01; Fig. 1B), respectively. In independent validation cohorts, the level of EVP concentration was higher in CRLM patients compared to BD patients (*p* = 0.04; Fig. 1C) and was negatively associated with OS (*p* = 0.001; Fig. 1D). Using the median EVP concentration as a cut-off, high EVP concentration was associated with significantly decreased median survival in the discovery (before surgery: *p* < 0.001; after surgery: *p* = 0.0021) and the validation cohorts (*p* = 0.001; Fig. 1B, D), indicative of a prognostic value of EVP concentration in CRLM patients.

**Table 1:** Clinical characteristics of the entire study cohort

	Overall	Discovery cohort		Internal validation cohort		External validation cohort
		CRLM	BD	CRLM	BD	CRLM
n	405	56	7	154	78	110
Median follow-up time (days)	1253	1302		1633		831
Age (median)	66	61	60	65	66.5	68
Gender (%)						
Female	145 (35.8)	26 (46.4)	3 (42.9)	47 (30.5)	31 (40.0)	38 (34.5)
Male	260 (64.2)	30 (53.6)	4 (57.1)	107 (69.5)	47 (60.0)	72 (65.5)
Residual disease (%)						
No	294 (91.9)	52 (92.9)		135 (87.7)		107 (97.3)
Yes	26 ( 8.1)	4 (7.1)		19 (12.3)		3 (2.7)
Neoadjuvant therapy (%)						
No	76 (30.9)	4 (9.3)		50 (32.5)		22 (44.9)
Yes	170 (69.1)	39 (90.7)		104 (67.5)		27 (55.1)
Tumor differentiation (%)						
G1-G2	129 (83.2)	17 (73.9)		19 (63.3)		93 (91.2)
G3-G4	26 (16.8)	6 (26.1)		11 (36.7)		9 (8.8)
TNM (%)						
IVA	250 (78.1)	47 (83.9)		100 (64.9)		103 (93.6)
IVB	70 (21.9)	9 (16.1)		54 (35.1)		7 (6.4)
KRAS (%)						
Mutant	64 (41.3)	12 (30.8)		52 (44.8)		0 (0.0)
Wildtype	91 (58.7)	27 (69.2)		64 (55.2)		0 (0.0)
MSI (%)						
MSI-L	9 (8.9)	4 (36.4)		5 (21.7)		0 (0.0)
MSI-H	92 (91.1)	7 (63.6)		18 (78.3)		67 (100.0)

Mass spectrometry (MS) is emerging as a valuable tool to gain insight into the biology and clinical utility of EVPs [11–13]. Here, we first examined the EVP composition in liquid biopsies of patients with CRLM using LC–MS with a cEV median particle size of 98.2 and a mean particle concentration of 3.5E + 11 (Supplementary Fig. 1A – D). A total of 563 proteins were detected in serum-derived EVs and most of the identified EVPs were involved in exosomes and extracellular components using Funrich software [14, 15] (Supplementary Fig. 1E). Statistical analysis revealed 76 proteins in patients with CRLM and 10 proteins in BD that differed significantly pre- and post-surgically (FDR < 0.05). Focussing on EVPs differentially expressed in CRLM only, 74 EVPs were uniquely changed (Fig. 1E). To assess whether these 74 EVPs as prognostic biomarkers in CRLM, univariate analyses were performed using each EVP as covariate. Thirty EVPs were pre-surgically associated with OS (*p* < 0.05) in the single biomarker model (Supplementary Table 1). Using the LASSO-Cox method six proteins to predict prognosis were identified (Supplementary Fig. 2A). For validation, ELISAs of these 6 EVPs were performed and significantly correlated to the proteomic data (Supplementary Fig. 2B). Using multivariate Cox regression, an EVP panel was constructed based on the ELISA data. Four of the six proteins were included (*p*-value < 0.05 Wald statistic, Supplementary Table 2) and a risk score was calculated. The high-risk group (risk score > -0.3316339) had 34 observations with 14 deaths in year 1, 24 deaths in year 2, 28 deaths in year 3; compared to the low-risk group (risk score ≥ -0.3316339) that had 22 observations with no deaths in year 1, one death in year 2 and four deaths in year 3. The number of deaths after surgical resection was significantly higher after 1 year (*p* < 0.002), 2 years (*p* < 0.001) and 3 years (*p* < 0.001; Supplementary Fig. 3A) in the high-risk group. Kaplan–Meier analyses revealed a statistical difference in the median survival of the high-risk and low-risk groups (*p* < 0.0001; Fig. 1F). Some of these four proteins have been previously linked to CRC progression, such as the monocyte marker CD14 [16] and Serpin A4, a regulator of angiogenesis [17], whilst little is known about the role of CFP, a positive regulator of the complement system, and LBP (lipopolysaccharide binding protein). The risk score was also evaluated to the independent validation cohorts (Table 1) for the number of deaths in year 1, 2 and 3 in the internal cohort (*p* < 0.002, *p* < 0.0001, *p* < 0.0001; Supplementary Fig. 3B), and external cohort (*p* = 0.13, *p* = 0.07, *p* = 0.03; Supplementary Fig. 3C). Kaplan–Meier curves of the validation cohorts indicated that the EVP signature could distinguish between the risk of recurrence and death of CRLM patients (internal: p < 0.0001 and external: *p* = 0.004 validation cohort; Fig. 1G, H). The revealed AUC values of the EVP signature between year 1 and year 4 were higher compared to the TNM for each cohort (Fig. 1I-K). The risk score was associated with MSI status, neoadjuvant therapy and TNM stage (all *p* < 0.05, Supplementary Table 3). An increased risk score in MSI-H, no neoadjuvant therapy and TNM stage IVB-IVC was observed (Supplementary Fig. 4a-c). However, the signature in specific subgroups was still of high prognostic accuracy (all *p* < 0.05; Supplementary Fig. 4d-e**)**.

To decipher a robust signature, the CRLM patients were divided into training and test set, with a median follow-up time of 40.1 and 42.8 months (Supplementary Table 4), respectively. Without compromising the discriminative ability, MSI status and tumour differentiation were excluded due to incomplete data. Age (> Median (66 years); *p* = 0.006), EVP signature (High-risk; *p* < 0.0001), EVP concentration (≥ Median (54.5 ug/ul); *p* < 0.001) and TNM (*p* = 0.001) were independent prognostic factors for OS (Supplementary Table 5) and integrated into a prognostic nomogram (Supplementary Fig. 5a) revealing that the EVP signature was the largest contributor to prognosis. The calibration plots aligned well in the training and test set between the predictive power of the nomogram and actual observation (Supplementary Fig. 5b). The C-index of the training set for OS prediction (0.78; 95% CI:0.74–0.83) was significantly higher than the model comprised of age, EVP concentration and TNM stage (0.72; 95% CI:0.65–0.78; *p* = 0.003). A similar trend was observed in the test set with the C-index significantly greater for the nomogram prediction (0.82; 95% CI:0.77–0.87) than the model without the risk group stratification (0.76; 95% CI:0.69–0.83; *p* = 0.008). The cut-off values were determined by splitting the patients into three subgroups CRLM1, CRLM2, and CRLM3, each representing a distinct prognosis after sorting by total point in the training set. Applying the cut-off values to the test set allowed for significant differences in survival using Kaplan–Meier analyses (Supplementary Fig. 5c and d).

### EV-bound CXCL7 predicts early response to chemotherapy

**Fig. 2:**
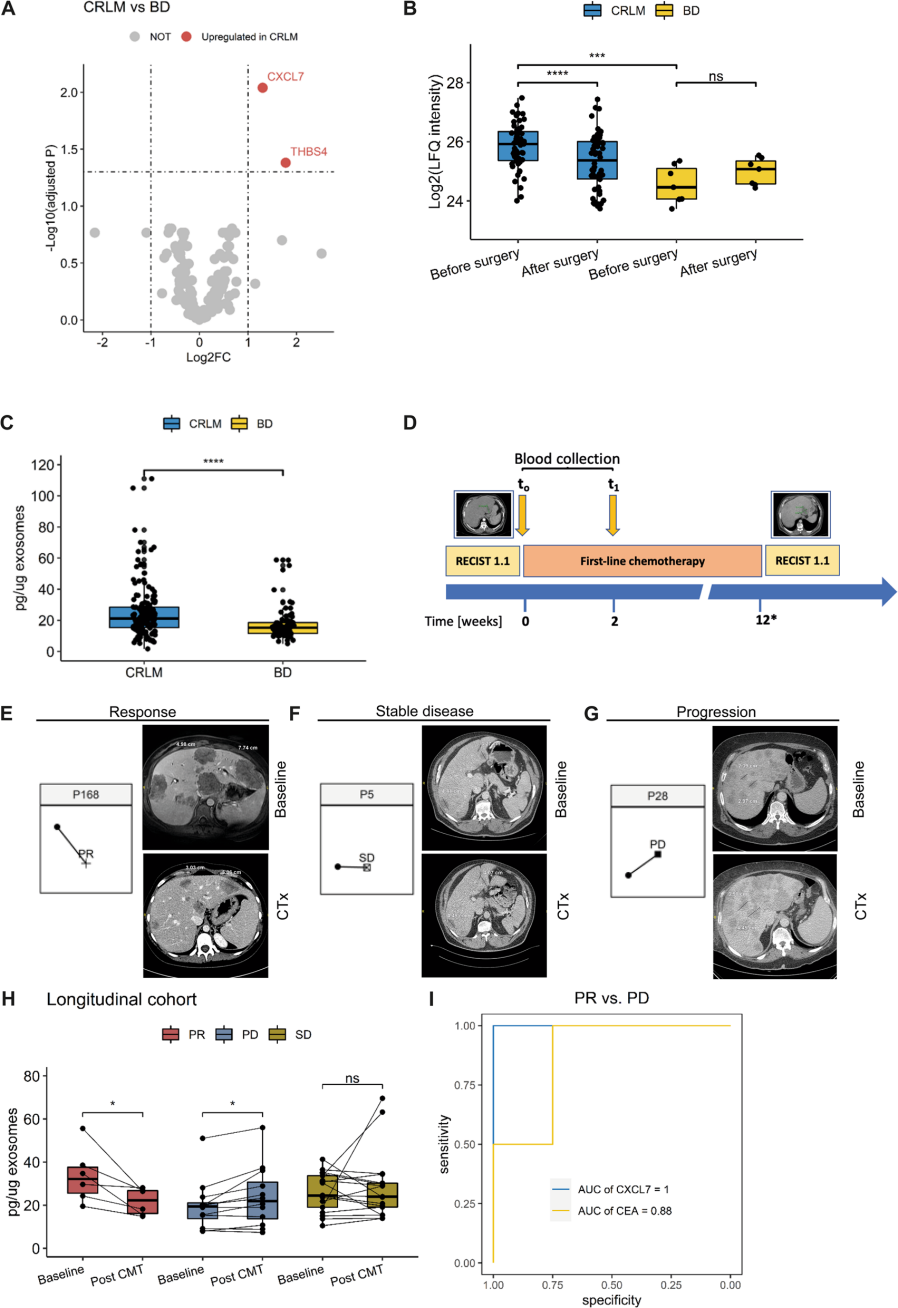
**A** Volcano plot of differently expressed (FDR < 0.05 ) EVPs between CRLM and BD in the discovery cohort using mass spectrometry. Each dot represents an individual protein. Boxplot analysis of EVP CXCL7 in the discovery cohort (**B**), and in the internal validation cohort (**C**) ns: p>0.05,***: *p*≤0.001, ****: *p*≤0.0001. p values by Mann-Whitney U test. **D** Scheme of longitudinal blood sampling before the start of chemotherapy and two weeks after the start of chemotherapy. Imaging by CT-scan was performed before CMT and 3 months (median range: 1 – 9 months) after the start of CMT. (E-G) Three representative cases of patients either with a decrease of EV-bound CXCL7 and partial response (**E**), or with stable EV-bound CXCL7 and stable disease (**F**) or with increase of EV-bound CXCL7 and progressive disease (**G**). **H** Boxplot analysis of EVP CXCL7 in the longitudinal cohort for chemotherapy respons prediction. CR: complete response, PR: partial response, SD: stable disease, PD: progressive disease. ns: *p*>0.05,***: *p*≤0.001, ****: *p*≤0.0001. *p* values by Mann-Whitney U test. **I**: ROC curve analysis for prediction of CR/PR vs PD after completion of first round of chemotherapy.

For identifying cancer-specific EVPs, the proteomes of CRLM and BD were compared prior to surgery applying following criteria: EVPs present in > 50% of the samples, and, of those, a > twofold increase compared to BD with a FDR of < 0.05. Chemokine ligand 7 (CXCL7) and Thrombospondin 4 (THBS4) were identified as CRLM-enriched EV-bound proteins (Fig. 2A). CXCL7 was investigated in more detail as the CXCL7/CXCR2 axis has been implied to be a predictive marker of poor survival in metastatic CRC as well as a diagnostic serum marker in CRC [18, 19]. Moreover, only CXCL7 was downregulated after tumour removal with > twofold and FDR < 0.05 (Fig. 2B), suggesting metastatic lesions as major source of EV-bound CXCL7. The validation cohort showed a similar observation of the CXCL7 levels in CRLM compared to BD (*p* < 0.0001; Fig. 2C) and the ROC analysis revealed an AUC of 0.708 (0.617–0.799) and 0.764 (0.686–0.842) using CXCL7 alone or in combination with the EVP concentration, respectively (Supplementary Fig. 6). This prompted us to determine, if EV-bound CXCL7 can also predict response to chemotherapy in CRLM patients. Longitudinal serum samples were collected from 35 patients undergoing chemotherapy (Supplementary Table 6) at baseline and 2 weeks after starting chemotherapy (Fig. 2D). Staging was performed after a median interval of 3 months (range: 1–6 months). Tumour response was assessed according to RECIST 1.1 [20] and correlated to the longitudinal EV-bound CXCL7 levels (Fig. 2E-H). All 6 patients with partial response(PR) according to the RECIST 1.1 criteria showed an early decrease in EV-bound CXCL7 levels already 2 weeks after the beginning of chemotherapy (Fig. 2E, H). Patients with a stable disease (SD) had no significant change of EV-bound CXCL7 (Fig. 2F, H). The majority of patients with progressive disease (PD) showed an increase in EV-bound CXCL7 two weeks after the beginning of chemotherapy (Fig. 2H). Prediction of PR vs PD by measured expression level of CXCL7 revealed a model with an AUC of 1.00 (95% CI:1.00–1.00) superior to the CEA level (Fig. 2I).

## Conclusion

In this study, we present a preoperative risk discrimination strategy for patients with resectable CRLM for postoperative disease recurrence and survival. Using matched pre- and post-operative serum samples of patients undergoing CRLM resection, we developed a signature of four EVPs with preoperative prognostic value in three independent cohorts. In addition to age and TNM stage, the EVP signature and EVP concentration were identified as independent prognostic factors. EV-bound CXCL7 was found as a biomarker of early response in CRLM patients receiving systemic chemotherapy.

## Supplementary Information

**Additional file 1.**

**Additional file 2.**

## Data Availability

The datasets used and/or analysed during the current study are available from the corresponding author on reasonable request.

## Abbreviations

AUC: Area under the curve
BD: Benign disease
C-index: Concordance index
CEA: Carcinoembryonic antigen
CI: Confidential interval
CXCL7: Chemokine (C-X-C motif) ligand 7
cEVs: Circulating extracellular vesicles
CRC: Colorectal cancer
CR LM: Colorectal liver metastases
ELISA: Enzyme-linked immunosorbent assay
EV: Extracellular vesicles
EVP: Extracellular vesicle proteins
FDR: False Discovery Rate
i.e.: Id est
LC–MS: Liquid Chromatography Mass Spectrometry
mRNA: Messenger ribonucleic acid
MSI: Microsatellite Instability
OS: Overall survival
PD: Progressive disease
PR: Partial response
RECIST: Response Evaluation Criteria In Solid Tumors
SD: Stable disease
THBS4: Thrombospondin 4
