# Supplementary material for: Comprehensive proteomic profiling of serum extracellular vesicles in patients with colorectal liver metastases identifies a signature for non-invasive risk stratification and early-response evaluation
Source: Mol Cancer. 2022 Apr 1;21:91. doi: 10.1186/s12943-022-01562-4 (PMC8973547; doi:10.1186/s12943-022-01562-4)
Supplement: Supplementary file 1 — Additional file 1. [file 12943_2022_1562_MOESM1_ESM.pptx]

## Slide 1
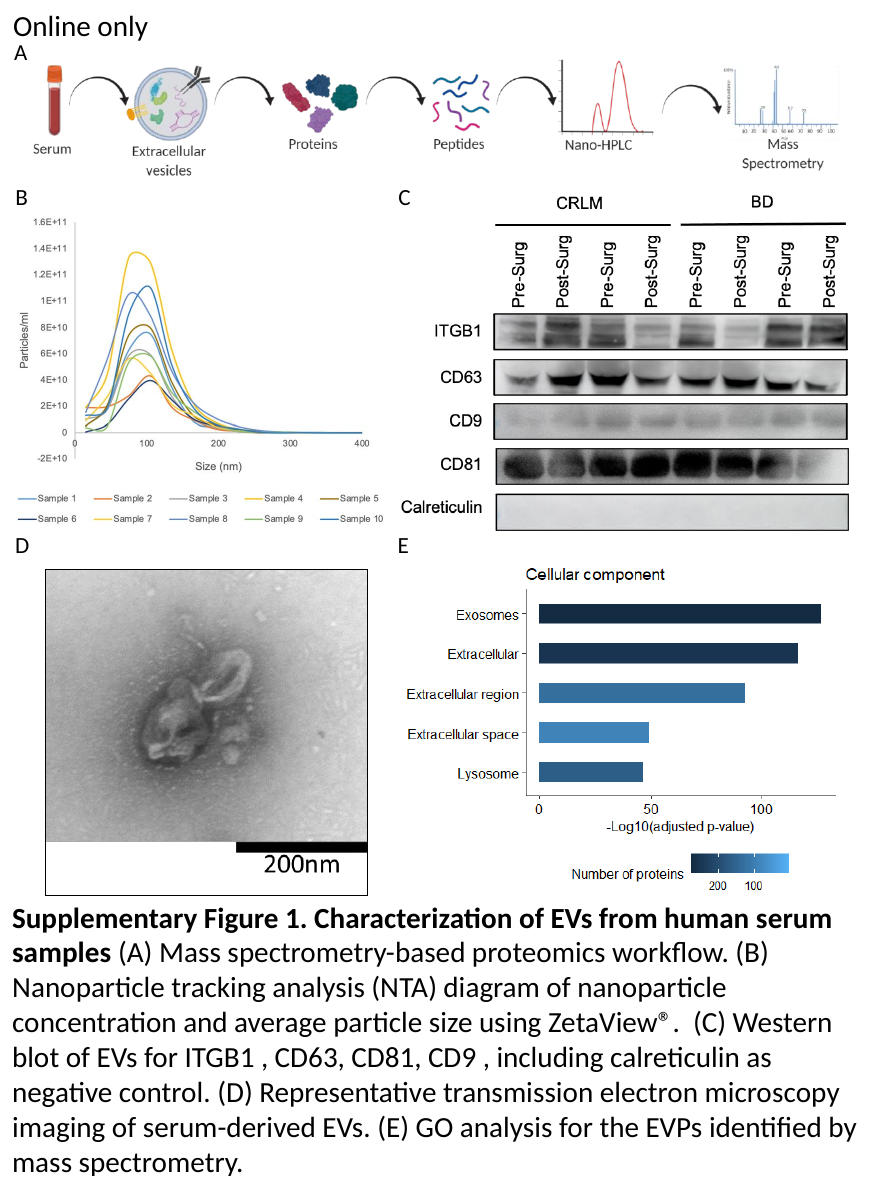

Online only
A
B
C
D
E
Supplementary Figure 1. Characterization of EVs from human serum samples (A) Mass spectrometry-based proteomics workflow. (B) Nanoparticle tracking analysis (NTA) diagram of nanoparticle concentration and average particle size using ZetaView®. (C) Western blot of EVs for ITGB1 , CD63, CD81, CD9 , including calreticulin as negative control. (D) Representative transmission electron microscopy imaging of serum-derived EVs. (E) GO analysis for the EVPs identified by mass spectrometry.

## Slide 2
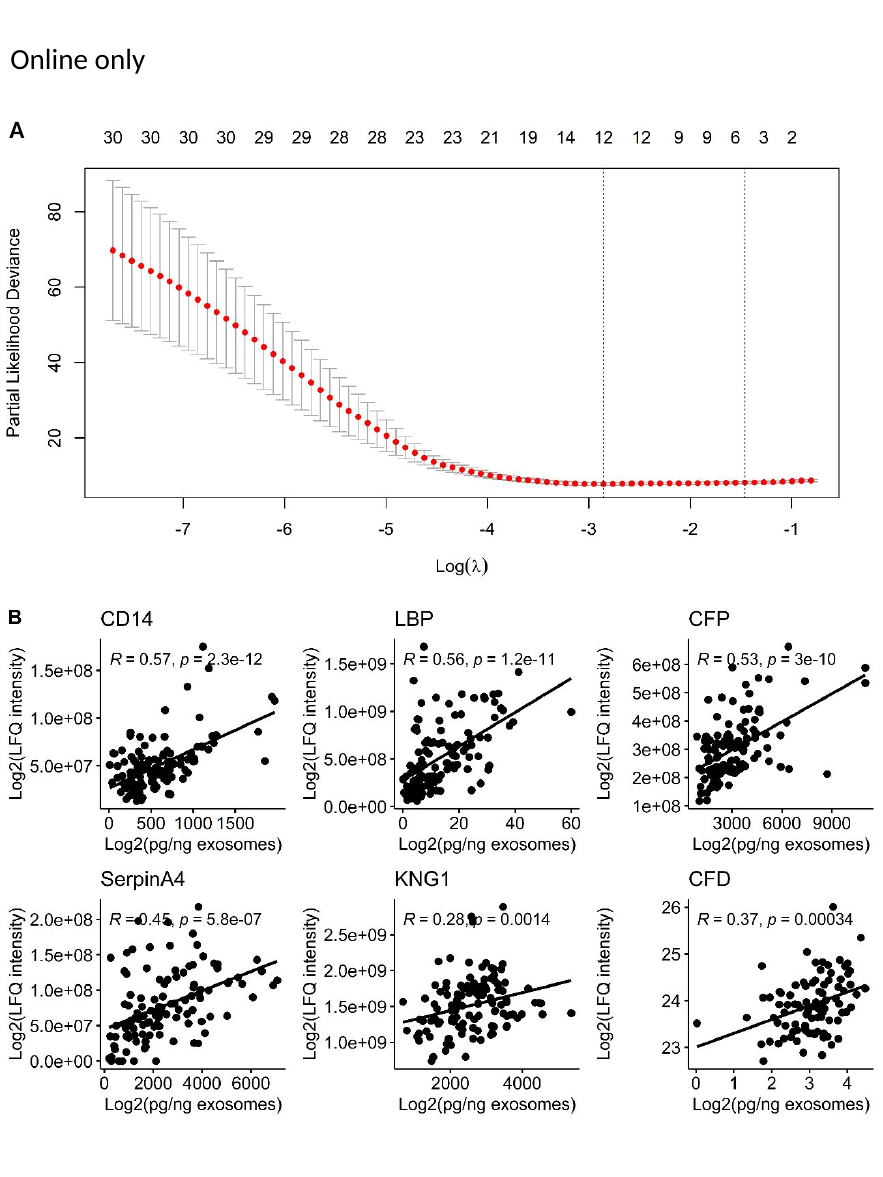

Online only
A

## Slide 3
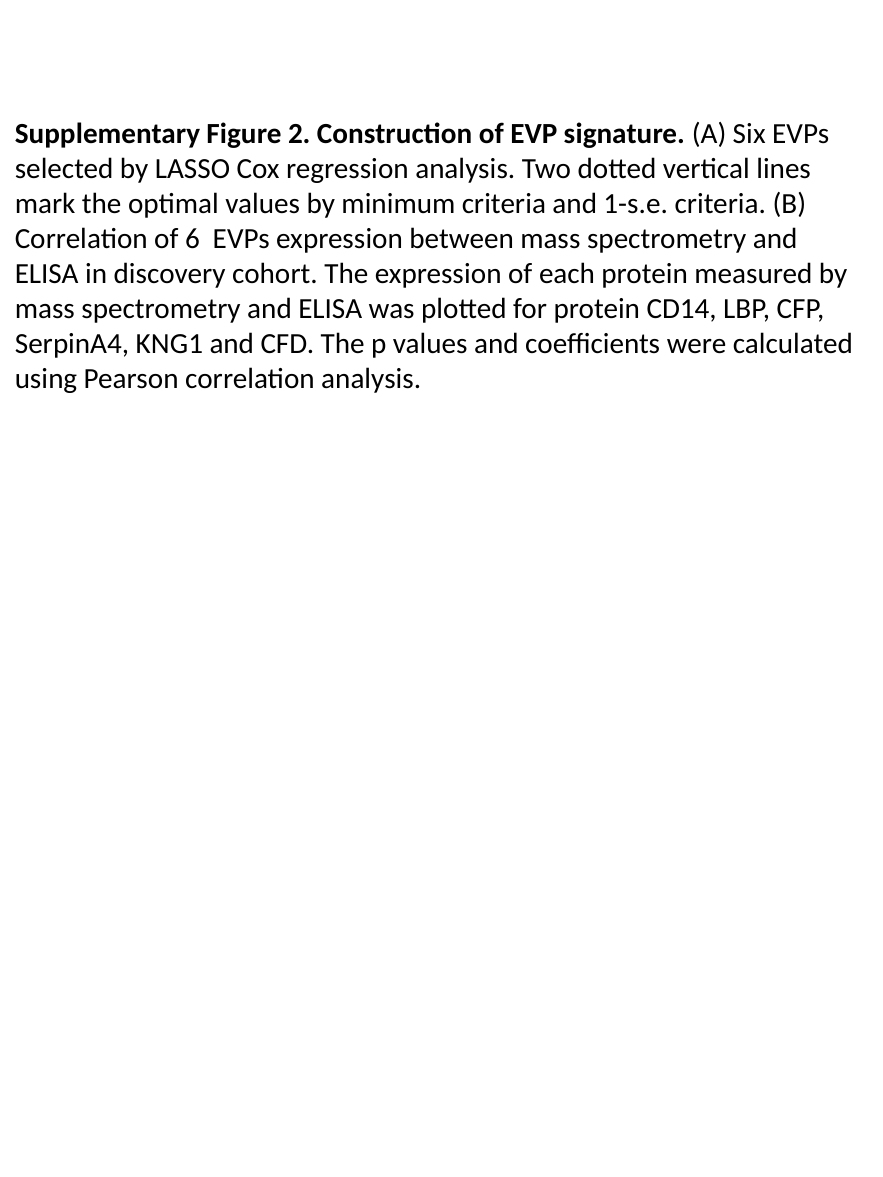

Supplementary Figure 2. Construction of EVP signature. (A) Six EVPs selected by LASSO Cox regression analysis. Two dotted vertical lines mark the optimal values by minimum criteria and 1-s.e. criteria. (B) Correlation of 6 EVPs expression between mass spectrometry and ELISA in discovery cohort. The expression of each protein measured by mass spectrometry and ELISA was plotted for protein CD14, LBP, CFP, SerpinA4, KNG1 and CFD. The p values and coefficients were calculated using Pearson correlation analysis.

## Slide 4
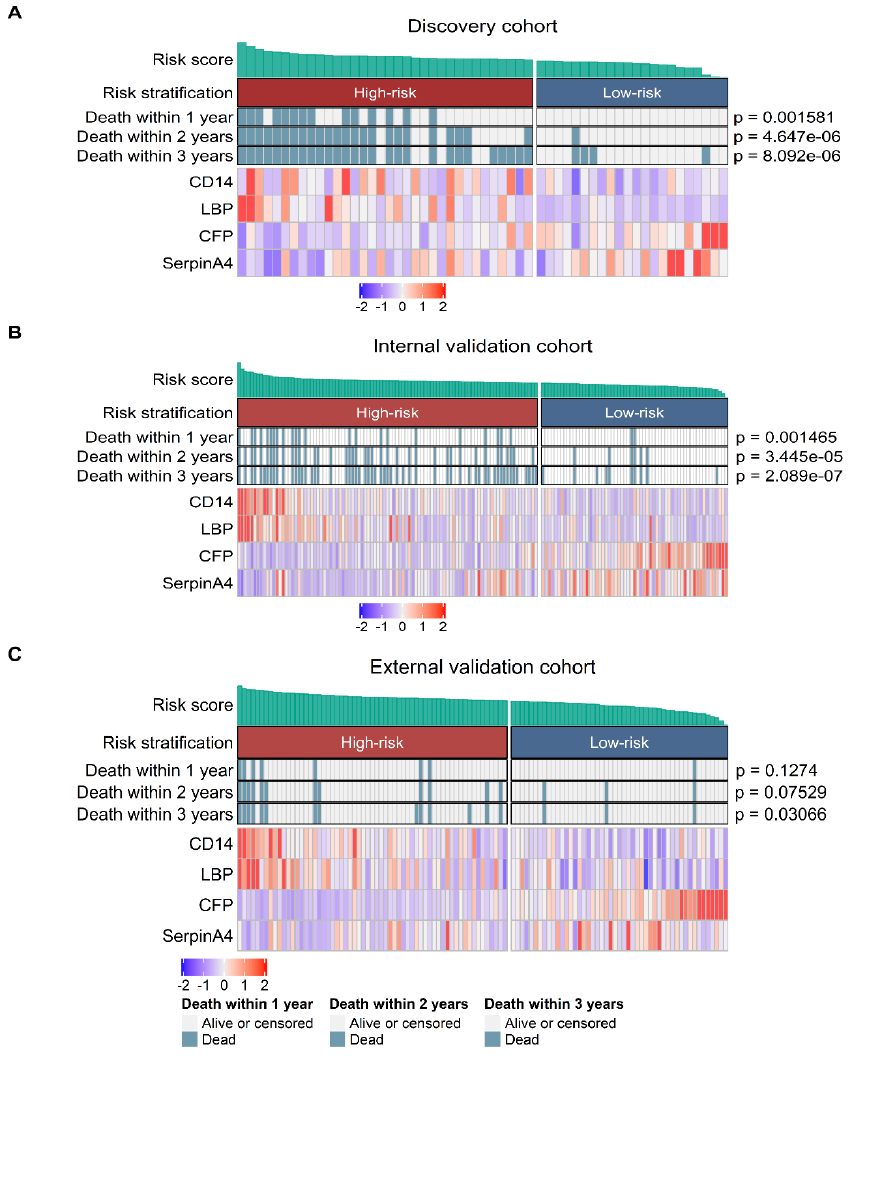

## Slide 5
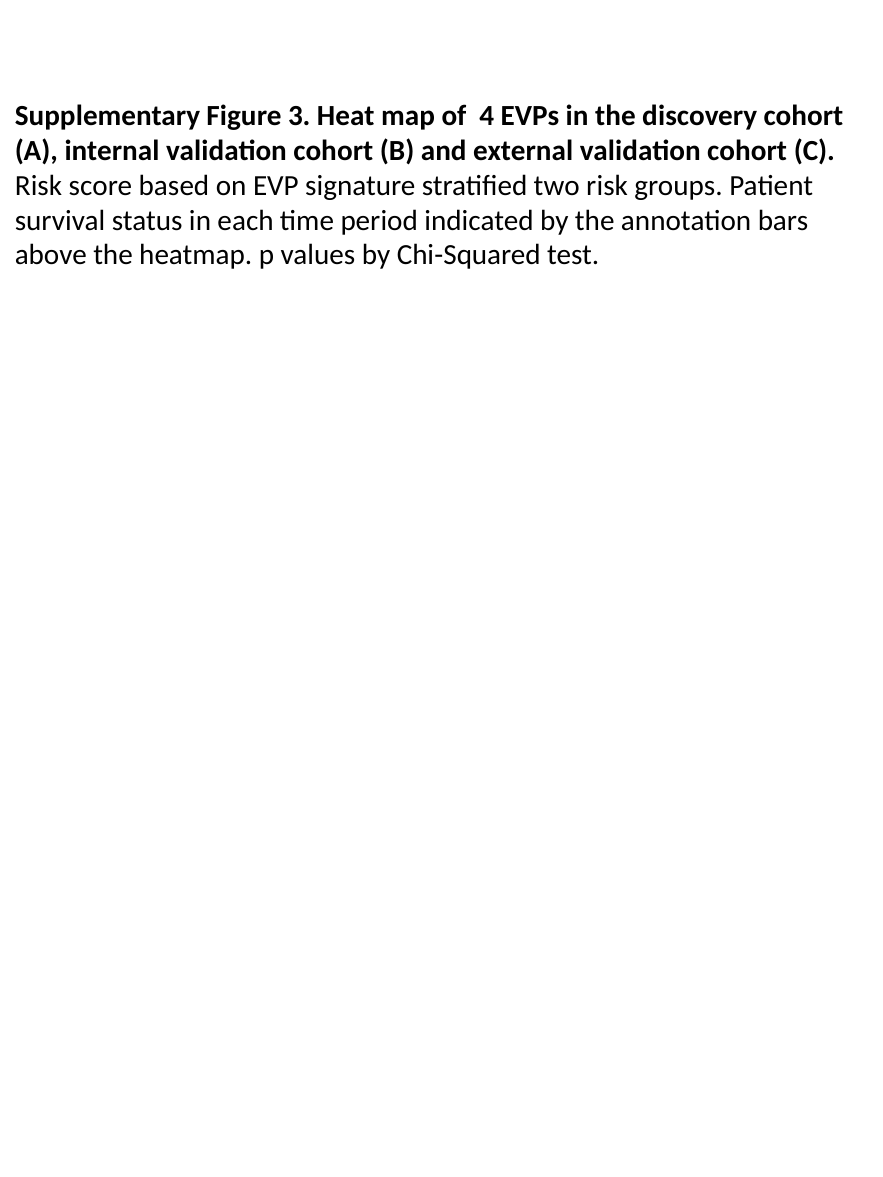

Supplementary Figure 3. Heat map of 4 EVPs in the discovery cohort (A), internal validation cohort (B) and external validation cohort (C). Risk score based on EVP signature stratified two risk groups. Patient survival status in each time period indicated by the annotation bars above the heatmap. p values by Chi-Squared test.

## Slide 6
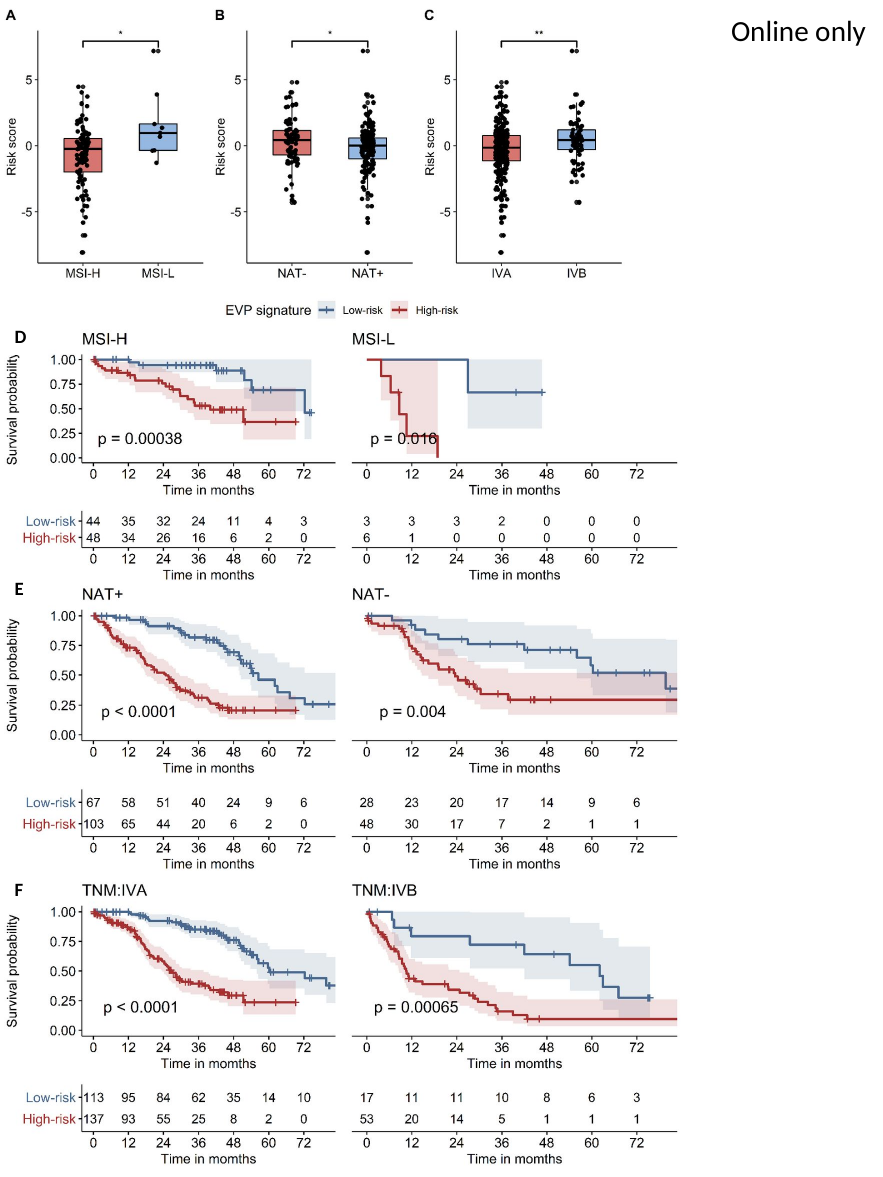

Online only
D
E
F

## Slide 7
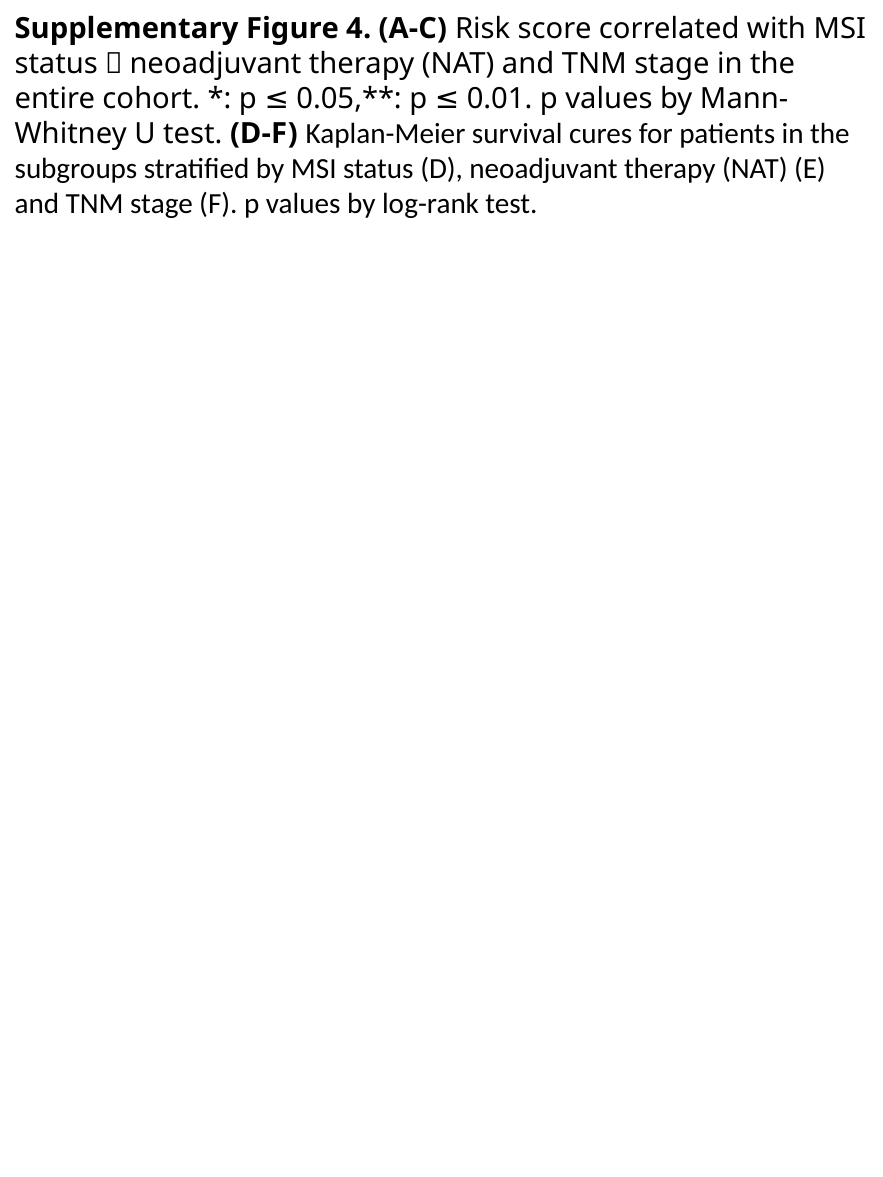

Supplementary Figure 4. (A-C) Risk score correlated with MSI status，neoadjuvant therapy (NAT) and TNM stage in the entire cohort. *: p ≤ 0.05,**: p ≤ 0.01. p values by Mann-Whitney U test. (D-F) Kaplan-Meier survival cures for patients in the subgroups stratified by MSI status (D), neoadjuvant therapy (NAT) (E) and TNM stage (F). p values by log-rank test.

## Slide 8
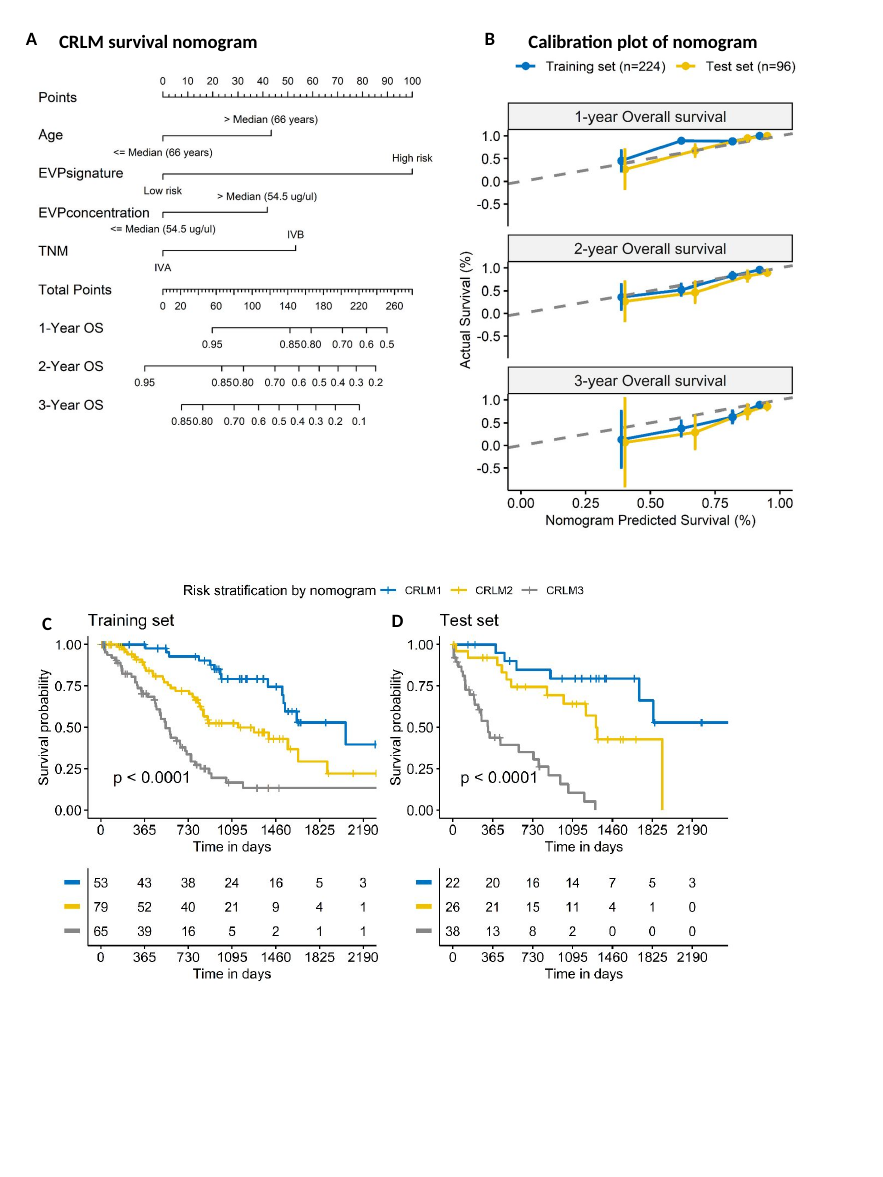

A
B
CRLM survival nomogram
Calibration plot of nomogram
D
C

## Slide 9
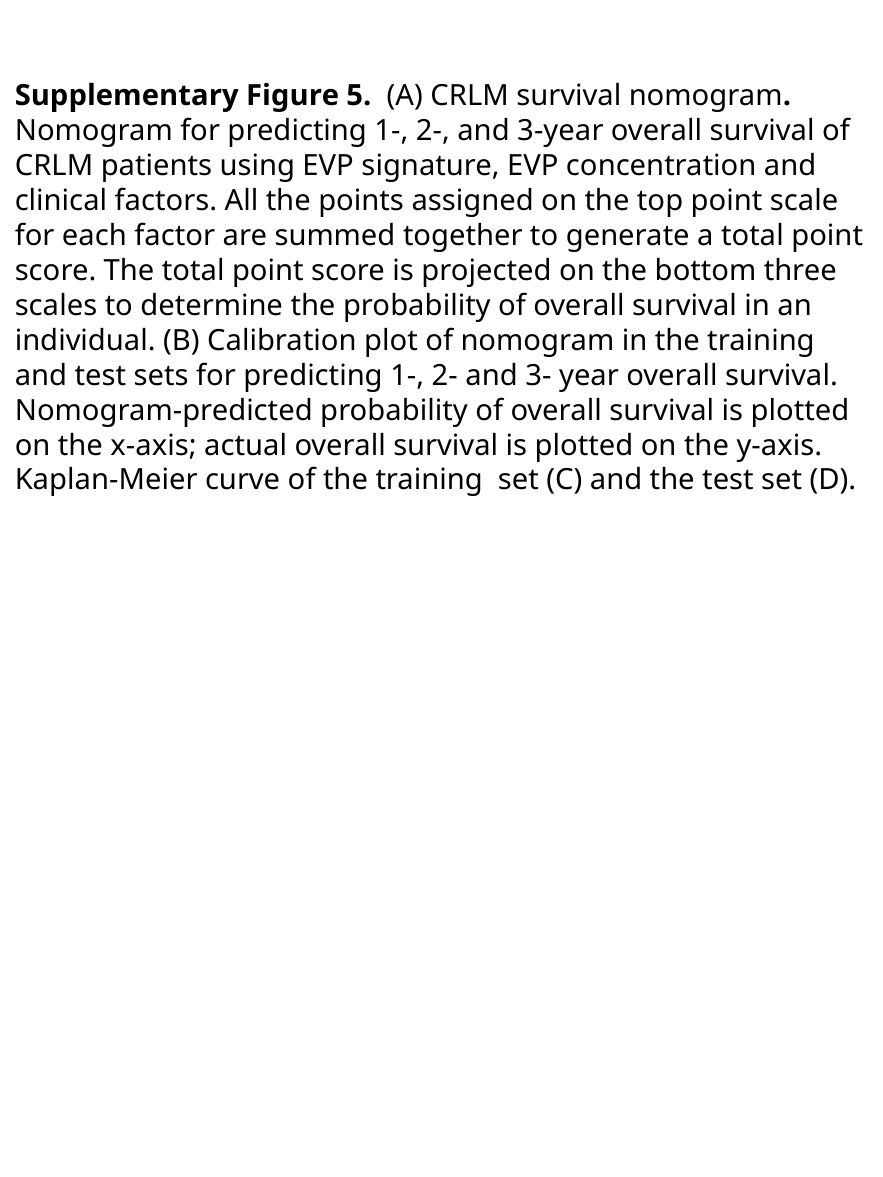

Supplementary Figure 5. (A) CRLM survival nomogram. Nomogram for predicting 1-, 2-, and 3-year overall survival of CRLM patients using EVP signature, EVP concentration and clinical factors. All the points assigned on the top point scale for each factor are summed together to generate a total point score. The total point score is projected on the bottom three scales to determine the probability of overall survival in an individual. (B) Calibration plot of nomogram in the training and test sets for predicting 1-, 2- and 3- year overall survival. Nomogram-predicted probability of overall survival is plotted on the x-axis; actual overall survival is plotted on the y-axis. Kaplan-Meier curve of the training set (C) and the test set (D).

## Slide 10
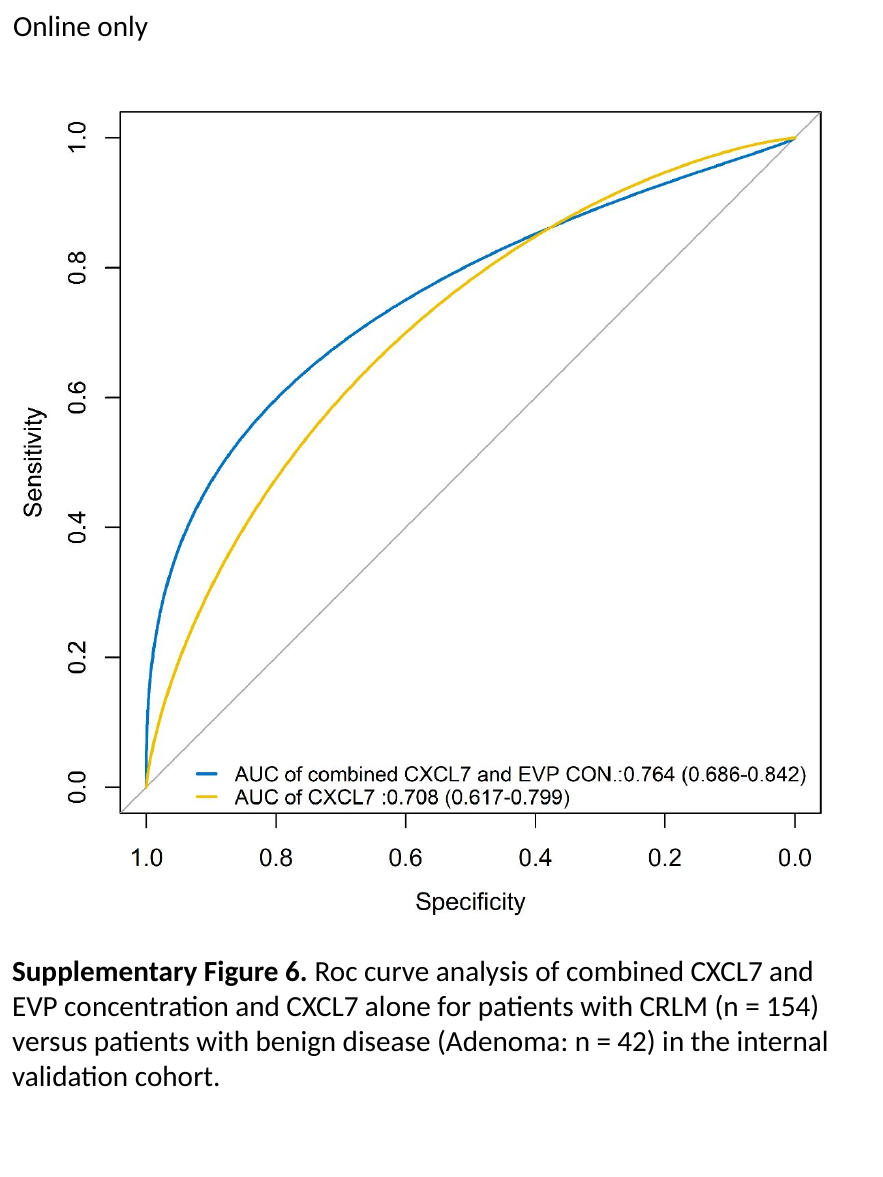

Online only
Supplementary Figure 6. Roc curve analysis of combined CXCL7 and EVP concentration and CXCL7 alone for patients with CRLM (n = 154) versus patients with benign disease (Adenoma: n = 42) in the internal validation cohort.
