# Supplementary material for: Comprehensive proteomic profiling of serum extracellular vesicles in patients with colorectal liver metastases identifies a signature for non-invasive risk stratification and early-response evaluation
Source: Mol Cancer. 2022 Apr 1;21:91. doi: 10.1186/s12943-022-01562-4 (PMC8973547; doi:10.1186/s12943-022-01562-4)
Supplement: Supplementary file 2 — Additional file 2. [file 12943_2022_1562_MOESM2_ESM.pptx]

## Slide 1
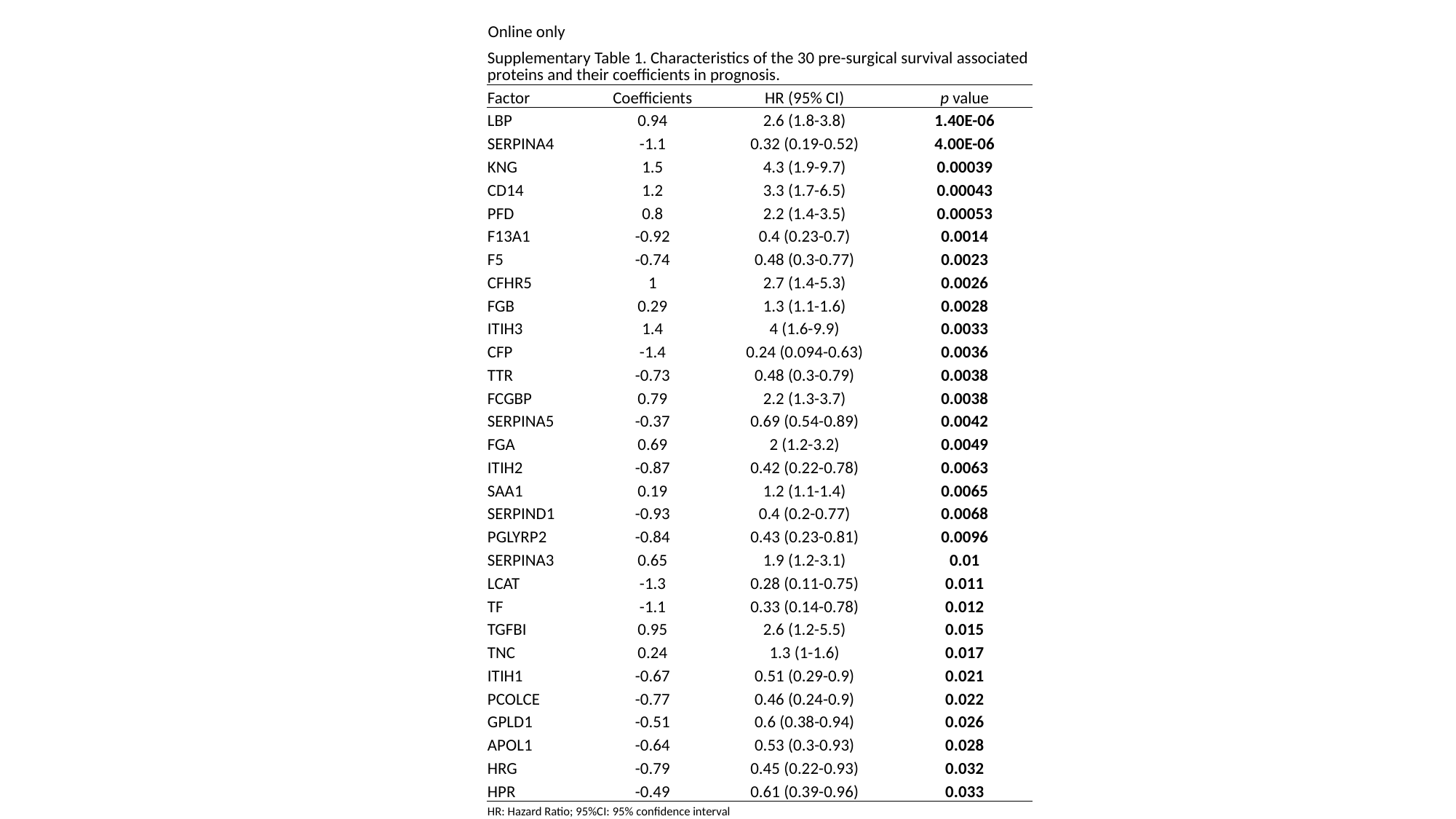

Online only
| Supplementary Table 1. Characteristics of the 30 pre-surgical survival associated proteins and their coefficients in prognosis. | | | | | | |
| --- | --- | --- | --- | --- | --- | --- |
| Factor | | Coefficients | | HR (95% CI) | | p value |
| LBP | | 0.94 | | 2.6 (1.8-3.8) | | 1.40E-06 |
| SERPINA4 | | -1.1 | | 0.32 (0.19-0.52) | | 4.00E-06 |
| KNG | | 1.5 | | 4.3 (1.9-9.7) | | 0.00039 |
| CD14 | | 1.2 | | 3.3 (1.7-6.5) | | 0.00043 |
| PFD | | 0.8 | | 2.2 (1.4-3.5) | | 0.00053 |
| F13A1 | | -0.92 | | 0.4 (0.23-0.7) | | 0.0014 |
| F5 | | -0.74 | | 0.48 (0.3-0.77) | | 0.0023 |
| CFHR5 | | 1 | | 2.7 (1.4-5.3) | | 0.0026 |
| FGB | | 0.29 | | 1.3 (1.1-1.6) | | 0.0028 |
| ITIH3 | | 1.4 | | 4 (1.6-9.9) | | 0.0033 |
| CFP | | -1.4 | | 0.24 (0.094-0.63) | | 0.0036 |
| TTR | | -0.73 | | 0.48 (0.3-0.79) | | 0.0038 |
| FCGBP | | 0.79 | | 2.2 (1.3-3.7) | | 0.0038 |
| SERPINA5 | | -0.37 | | 0.69 (0.54-0.89) | | 0.0042 |
| FGA | | 0.69 | | 2 (1.2-3.2) | | 0.0049 |
| ITIH2 | | -0.87 | | 0.42 (0.22-0.78) | | 0.0063 |
| SAA1 | | 0.19 | | 1.2 (1.1-1.4) | | 0.0065 |
| SERPIND1 | | -0.93 | | 0.4 (0.2-0.77) | | 0.0068 |
| PGLYRP2 | | -0.84 | | 0.43 (0.23-0.81) | | 0.0096 |
| SERPINA3 | | 0.65 | | 1.9 (1.2-3.1) | | 0.01 |
| LCAT | | -1.3 | | 0.28 (0.11-0.75) | | 0.011 |
| TF | | -1.1 | | 0.33 (0.14-0.78) | | 0.012 |
| TGFBI | | 0.95 | | 2.6 (1.2-5.5) | | 0.015 |
| TNC | | 0.24 | | 1.3 (1-1.6) | | 0.017 |
| ITIH1 | | -0.67 | | 0.51 (0.29-0.9) | | 0.021 |
| PCOLCE | | -0.77 | | 0.46 (0.24-0.9) | | 0.022 |
| GPLD1 | | -0.51 | | 0.6 (0.38-0.94) | | 0.026 |
| APOL1 | | -0.64 | | 0.53 (0.3-0.93) | | 0.028 |
| HRG | | -0.79 | | 0.45 (0.22-0.93) | | 0.032 |
| HPR | | -0.49 | | 0.61 (0.39-0.96) | | 0.033 |
| HR: Hazard Ratio; 95%CI: 95% confidence interval | | | | | | |

## Slide 2
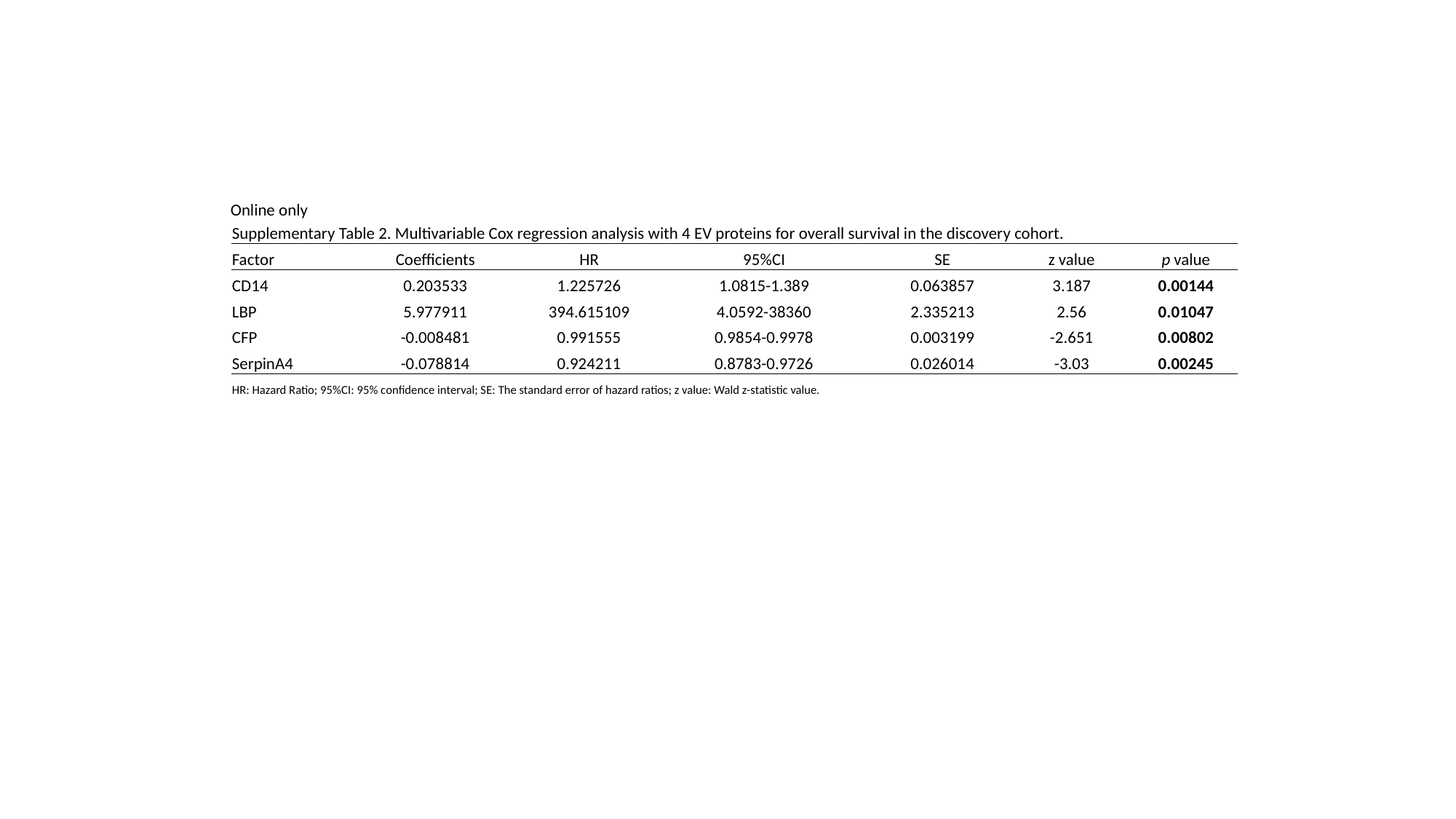

Online only
| Supplementary Table 2. Multivariable Cox regression analysis with 4 EV proteins for overall survival in the discovery cohort. | | | | | | | | | | | | |
| --- | --- | --- | --- | --- | --- | --- | --- | --- | --- | --- | --- | --- |
| Factor | | Coefficients | | HR | | 95%CI | | SE | | z value | | p value |
| CD14 | | 0.203533 | | 1.225726 | | 1.0815-1.389 | | 0.063857 | | 3.187 | | 0.00144 |
| LBP | | 5.977911 | | 394.615109 | | 4.0592-38360 | | 2.335213 | | 2.56 | | 0.01047 |
| CFP | | -0.008481 | | 0.991555 | | 0.9854-0.9978 | | 0.003199 | | -2.651 | | 0.00802 |
| SerpinA4 | | -0.078814 | | 0.924211 | | 0.8783-0.9726 | | 0.026014 | | -3.03 | | 0.00245 |
| HR: Hazard Ratio; 95%CI: 95% confidence interval; SE: The standard error of hazard ratios; z value: Wald z-statistic value. | | | | | | | | | | | | |

## Slide 3
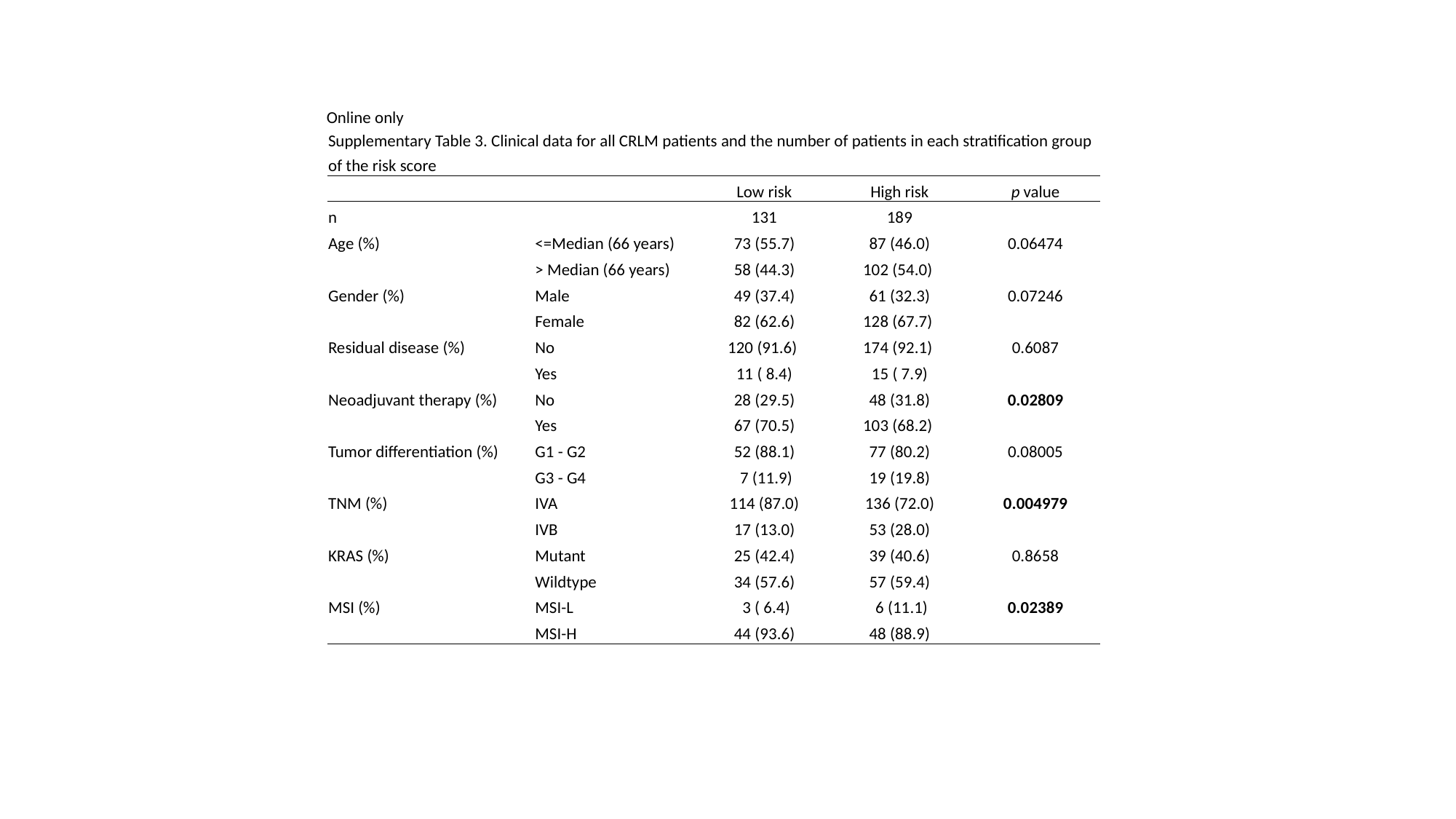

Online only
| Supplementary Table 3. Clinical data for all CRLM patients and the number of patients in each stratification group of the risk score | | | | |
| --- | --- | --- | --- | --- |
| | | Low risk | High risk | p value |
| n | | 131 | 189 | |
| Age (%) | <=Median (66 years) | 73 (55.7) | 87 (46.0) | 0.06474 |
| | > Median (66 years) | 58 (44.3) | 102 (54.0) | |
| Gender (%) | Male | 49 (37.4) | 61 (32.3) | 0.07246 |
| | Female | 82 (62.6) | 128 (67.7) | |
| Residual disease (%) | No | 120 (91.6) | 174 (92.1) | 0.6087 |
| | Yes | 11 ( 8.4) | 15 ( 7.9) | |
| Neoadjuvant therapy (%) | No | 28 (29.5) | 48 (31.8) | 0.02809 |
| | Yes | 67 (70.5) | 103 (68.2) | |
| Tumor differentiation (%) | G1 - G2 | 52 (88.1) | 77 (80.2) | 0.08005 |
| | G3 - G4 | 7 (11.9) | 19 (19.8) | |
| TNM (%) | IVA | 114 (87.0) | 136 (72.0) | 0.004979 |
| | IVB | 17 (13.0) | 53 (28.0) | |
| KRAS (%) | Mutant | 25 (42.4) | 39 (40.6) | 0.8658 |
| | Wildtype | 34 (57.6) | 57 (59.4) | |
| MSI (%) | MSI-L | 3 ( 6.4) | 6 (11.1) | 0.02389 |
| | MSI-H | 44 (93.6) | 48 (88.9) | |

## Slide 4
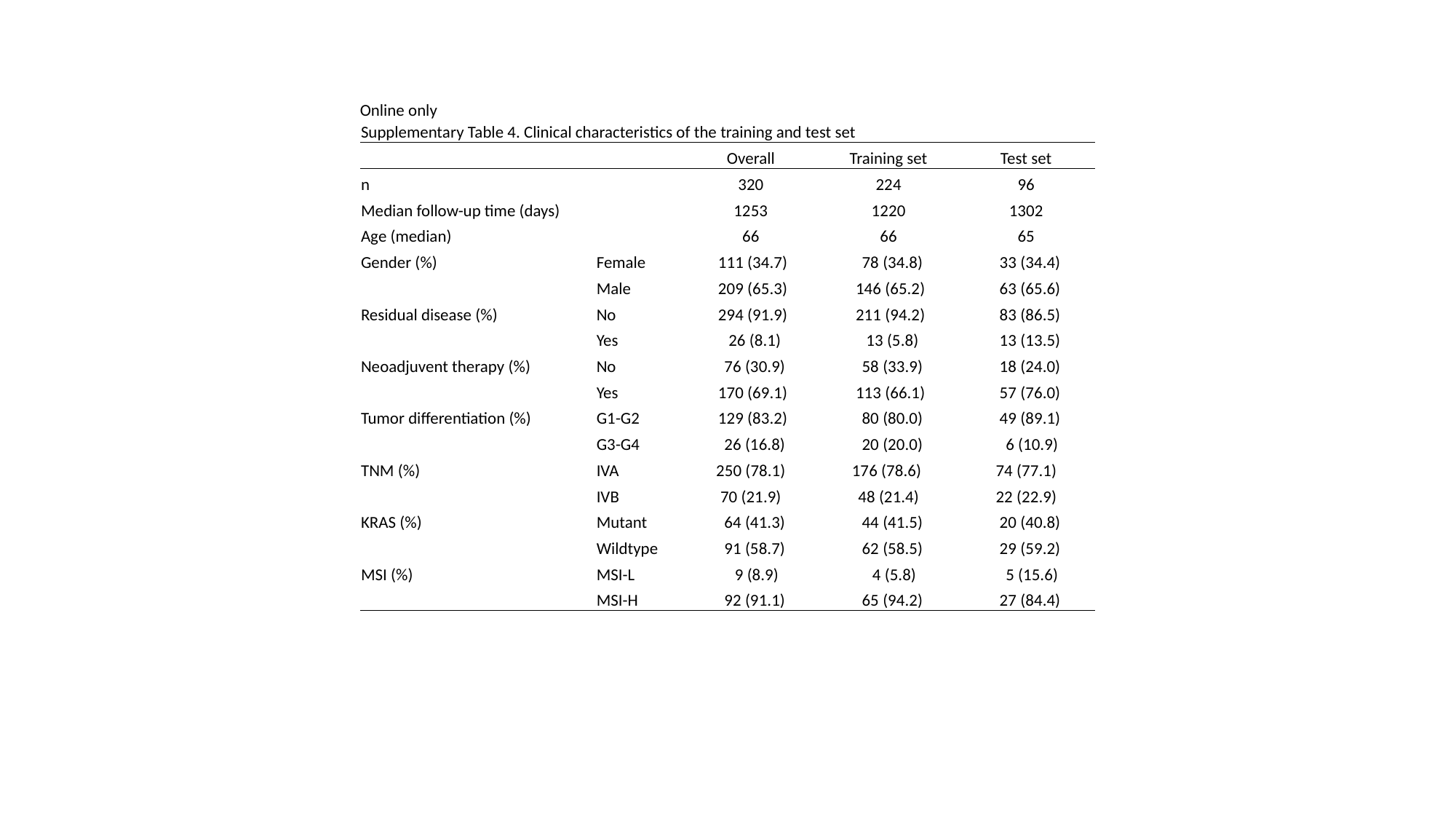

Online only
| Supplementary Table 4. Clinical characteristics of the training and test set | | | | |
| --- | --- | --- | --- | --- |
| | | Overall | Training set | Test set |
| n | | 320 | 224 | 96 |
| Median follow-up time (days) | | 1253 | 1220 | 1302 |
| Age (median) | | 66 | 66 | 65 |
| Gender (%) | Female | 111 (34.7) | 78 (34.8) | 33 (34.4) |
| | Male | 209 (65.3) | 146 (65.2) | 63 (65.6) |
| Residual disease (%) | No | 294 (91.9) | 211 (94.2) | 83 (86.5) |
| | Yes | 26 (8.1) | 13 (5.8) | 13 (13.5) |
| Neoadjuvent therapy (%) | No | 76 (30.9) | 58 (33.9) | 18 (24.0) |
| | Yes | 170 (69.1) | 113 (66.1) | 57 (76.0) |
| Tumor differentiation (%) | G1-G2 | 129 (83.2) | 80 (80.0) | 49 (89.1) |
| | G3-G4 | 26 (16.8) | 20 (20.0) | 6 (10.9) |
| TNM (%) | IVA | 250 (78.1) | 176 (78.6) | 74 (77.1) |
| | IVB | 70 (21.9) | 48 (21.4) | 22 (22.9) |
| KRAS (%) | Mutant | 64 (41.3) | 44 (41.5) | 20 (40.8) |
| | Wildtype | 91 (58.7) | 62 (58.5) | 29 (59.2) |
| MSI (%) | MSI-L | 9 (8.9) | 4 (5.8) | 5 (15.6) |
| | MSI-H | 92 (91.1) | 65 (94.2) | 27 (84.4) |

## Slide 5
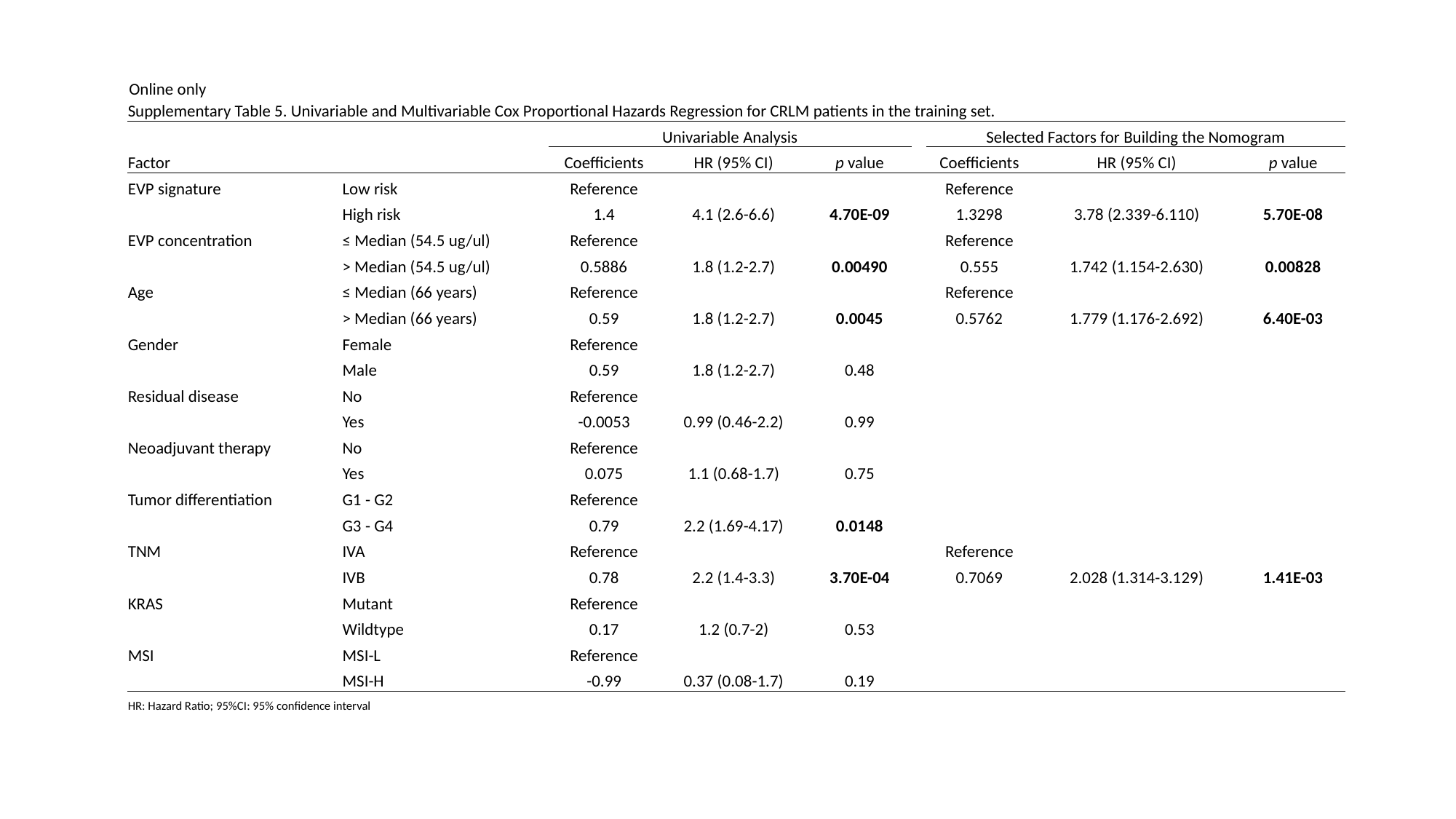

Online only
| Supplementary Table 5. Univariable and Multivariable Cox Proportional Hazards Regression for CRLM patients in the training set. | | | | | | | | | | | | |
| --- | --- | --- | --- | --- | --- | --- | --- | --- | --- | --- | --- | --- |
| | | Univariable Analysis | | | | | | Selected Factors for Building the Nomogram | | | | |
| Factor | | Coefficients | | HR (95% CI) | | p value | | Coefficients | | HR (95% CI) | | p value |
| EVP signature | Low risk | Reference | | | | | | Reference | | | | |
| | High risk | 1.4 | | 4.1 (2.6-6.6) | | 4.70E-09 | | 1.3298 | | 3.78 (2.339-6.110) | | 5.70E-08 |
| EVP concentration | ≤ Median (54.5 ug/ul) | Reference | | | | | | Reference | | | | |
| | > Median (54.5 ug/ul) | 0.5886 | | 1.8 (1.2-2.7) | | 0.00490 | | 0.555 | | 1.742 (1.154-2.630) | | 0.00828 |
| Age | ≤ Median (66 years) | Reference | | | | | | Reference | | | | |
| | > Median (66 years) | 0.59 | | 1.8 (1.2-2.7) | | 0.0045 | | 0.5762 | | 1.779 (1.176-2.692) | | 6.40E-03 |
| Gender | Female | Reference | | | | | | | | | | |
| | Male | 0.59 | | 1.8 (1.2-2.7) | | 0.48 | | | | | | |
| Residual disease | No | Reference | | | | | | | | | | |
| | Yes | -0.0053 | | 0.99 (0.46-2.2) | | 0.99 | | | | | | |
| Neoadjuvant therapy | No | Reference | | | | | | | | | | |
| | Yes | 0.075 | | 1.1 (0.68-1.7) | | 0.75 | | | | | | |
| Tumor differentiation | G1 - G2 | Reference | | | | | | | | | | |
| | G3 - G4 | 0.79 | | 2.2 (1.69-4.17) | | 0.0148 | | | | | | |
| TNM | IVA | Reference | | | | | | Reference | | | | |
| | IVB | 0.78 | | 2.2 (1.4-3.3) | | 3.70E-04 | | 0.7069 | | 2.028 (1.314-3.129) | | 1.41E-03 |
| KRAS | Mutant | Reference | | | | | | | | | | |
| | Wildtype | 0.17 | | 1.2 (0.7-2) | | 0.53 | | | | | | |
| MSI | MSI-L | Reference | | | | | | | | | | |
| | MSI-H | -0.99 | | 0.37 (0.08-1.7) | | 0.19 | | | | | | |
| HR: Hazard Ratio; 95%CI: 95% confidence interval | | | | | | | | | | | | |

## Slide 6
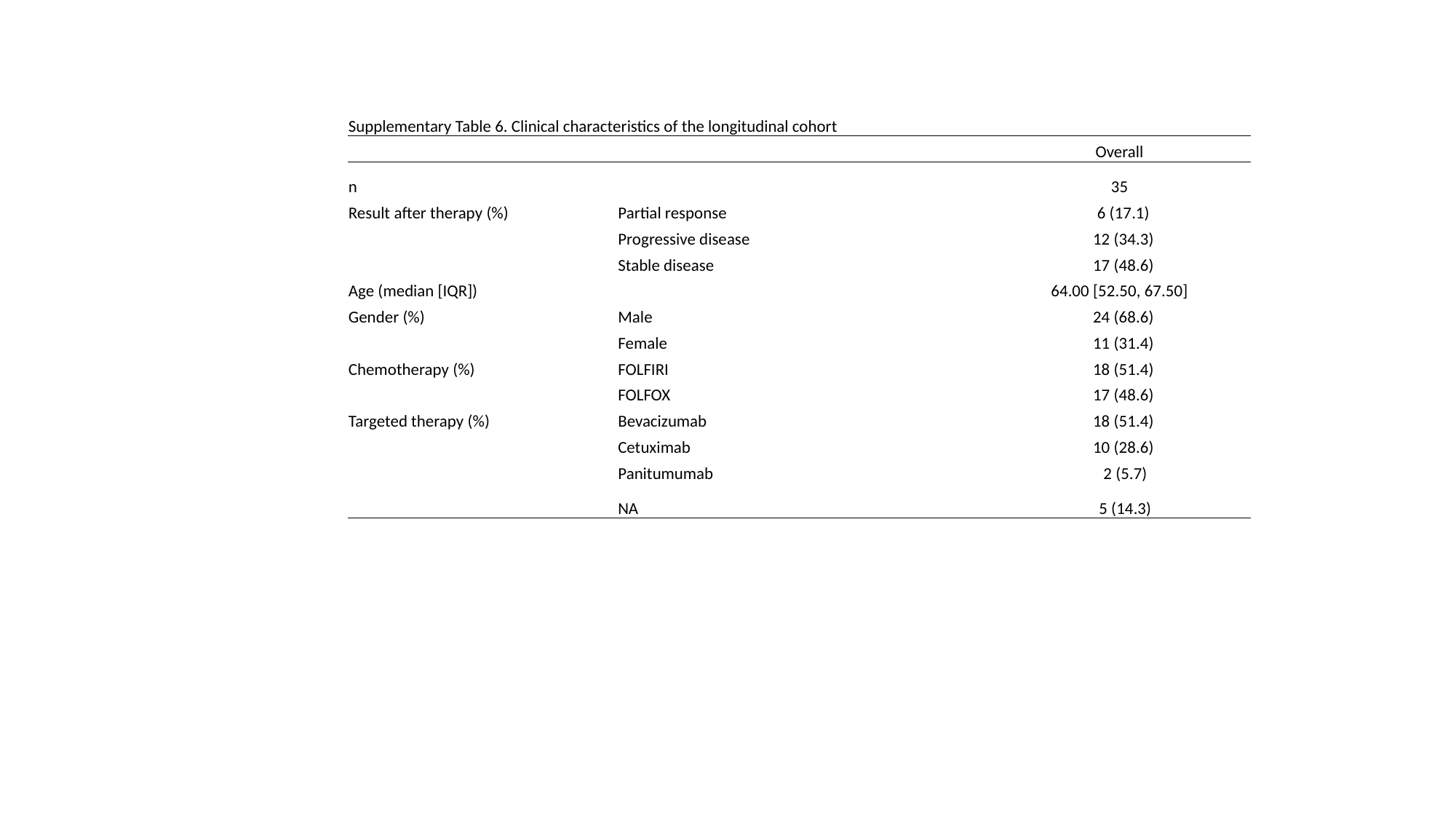

| Supplementary Table 6. Clinical characteristics of the longitudinal cohort | | |
| --- | --- | --- |
| | | Overall |
| n | | 35 |
| Result after therapy (%) | Partial response | 6 (17.1) |
| | Progressive disease | 12 (34.3) |
| | Stable disease | 17 (48.6) |
| Age (median [IQR]) | | 64.00 [52.50, 67.50] |
| Gender (%) | Male | 24 (68.6) |
| | Female | 11 (31.4) |
| Chemotherapy (%) | FOLFIRI | 18 (51.4) |
| | FOLFOX | 17 (48.6) |
| Targeted therapy (%) | Bevacizumab | 18 (51.4) |
| | Cetuximab | 10 (28.6) |
| | Panitumumab | 2 (5.7) |
| | NA | 5 (14.3) |
